# Vascular Remodeling in Rosacea: A Study on Microcirculatory Insights Using Oral Capillaroscopy

**DOI:** 10.1111/jocd.70012

**Published:** 2025-02-07

**Authors:** Abdullah Demirbas, Gozde Ulutaş Demirbas, Esin Diremsizoglu

**Affiliations:** ^1^ Department of Dermatology Kocaeli University Faculty of Medicine İzmit Kocaeli Turkey; ^2^ Department of Dermatology Kocaeli City Hospital İzmit Kocaeli Turkey

**Keywords:** capillaroscopy, microcirculation, oral capillaroscopy, rosacea

## Abstract

**Background:**

Rosacea is a chronic inflammatory skin condition characterized by facial erythema, telangiectasia, and papules. Although clinical assessment is essential for diagnosis, objective criteria for evaluating severity are lacking. This study aimed to investigate the relationship between rosacea severity, disease duration, and associated microvascular changes through oral mucosal capillaroscopy.

**Methods:**

This cross‐sectional case–control study included patients with rosacea and healthy controls. Oral capillaroscopy was performed to evaluate capillary morphology, analyzing parameters such as capillary arrangement (regular or irregular), presence of dot vessels, microhemorrhages, glomerular vessels, megacapillaries, tortuous vessels, areas of discoloration, and hyperkeratosis.

**Results:**

A total of 100 patients diagnosed with rosacea and 100 healthy controls were included in the study. Oral capillaroscopic findings revealed significantly higher rates of family history of rosacea and tortuous capillaries in the patient group. Patients with microhemorrhages exhibited a longer disease duration. No significant differences in tortuous capillary positivity were found between the papulopustular and erythematotelangiectatic subtypes; however, phymatous rosacea demonstrated no positive findings. Additionally, moderate‐severity patients had lower rates of microhemorrhage positivity, whereas the presence of tortuous capillaries increased with severity.

**Conclusion:**

Oral mucosal capillaroscopy is a valuable tool for assessing microvascular damage in rosacea and may serve as a diagnostic and prognostic marker for effective management.

## 1 Introduction

Rosacea is a chronic inflammatory skin condition characterized by facial erythema, telangiectasia, papules, and pustules, affecting approximately 5%–10% of the population, particularly in fair‐skinned individuals. The exact mechanisms underlying its pathophysiology remain unclear. Genetic studies suggest a hereditary component, with specific mutations, such as those in the cathelicidin antimicrobial peptide gene, linked to the inflammatory responses observed in rosacea [1, 2]. Inflammatory mediators, including nitric oxide, cathelicidins, and vascular endothelial growth factor (VEGF), contribute to vasodilation and angiogenesis in affected individuals. VEGF, in particular, is a key regulator of pathological angiogenesis and increased vascular permeability, which are associated with persistent erythema and telangiectasias. This increase in angiogenesis and permeability may lead to microvascular changes on the skin and mucosal surfaces [3, 4, 5]. Environmental factors, such as UV exposure, temperature changes, humidity, and dietary triggers (spicy foods, alcohol), are known to aggravate rosacea. Stress and hormonal fluctuations may also contribute to flare‐ups. These factors can lead to increased flushing and inflammation in susceptible individuals, emphasizing the importance of microvascular changes and inflammation in the pathophysiology of rosacea [6]. Parameters such as papules, pustules, erythema, and telangiectasias are evaluated by physicians and patients, but a more objective, consistent tool for assessing disease progression is needed [7, 8].

Capillaroscopy allows detailed imaging of the peripheral microvasculature, providing insights into microvascular damage, which is critical for both diagnosis and follow‐up [9, 10, 11, 12]. Oral capillaroscopy, a non‐invasive, simple, and risk‐free method, has emerged as a valuable tool in assessing microvascular abnormalities, particularly within the scope of autoimmune and chronic inflammatory diseases [13, 14, 15, 16, 17, 18, 19, 20]. By enabling the direct examination of capillary structures on mucosal surfaces, oral capillaroscopy also facilitates observation of the impact of VEGF on these microvascular changes. Therefore, investigating microvascular changes in rosacea through oral capillaroscopy may contribute to understanding VEGF‐mediated pathological processes and offer a useful tool for assessing disease severity.

This study aimed to investigate the relationship between rosacea severity, disease duration, and associated microvascular changes, specifically through an analysis of oral capillaroscopic findings using dermoscopy.

## 2 Methods

This cross‐sectional case–control study was conducted at a dermatology outpatient clinic, including patients diagnosed with rosacea and matched healthy controls. Rosacea diagnoses were confirmed by dermatologists based on established clinical criteria, while controls were individuals without any known dermatological conditions.

Inclusion criteria required adult patients diagnosed with any rosacea subtype, with severity categorized as mild, moderate, or severe according to the National Rosacea Society. Exclusion criteria included oral mucosal pathologies (such as candidiasis, lichen planus, glossitis, and periodontitis), age under 18, smoking, medications affecting microvascular structures (antidiabetics, antihypertensives, antilipidemics, thyroid medications), pregnancy or breastfeeding, and systemic disorders including diabetes mellitus, hypertension, cardiovascular diseases, hyperthyroidism, hypothyroidism, Sjögren syndrome, systemic lupus erythematosus, scleroderma, dermatomyositis, mixed connective tissue disease, or antiphospholipid syndrome. Data collected included age, gender, Fitzpatrick skin phototype, family history of rosacea, rosacea stage, disease duration, and previous treatments.

**FIGURE 1:**
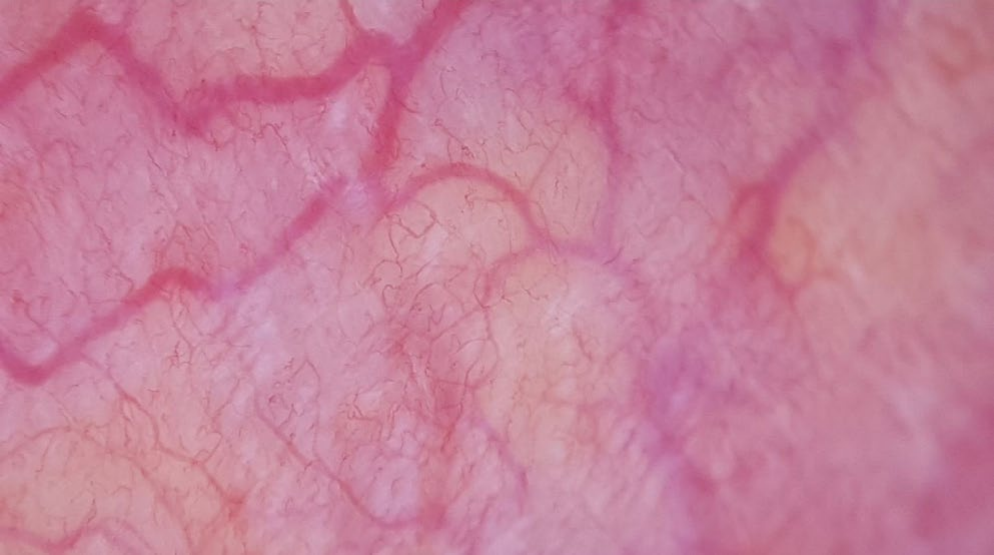
Regular capillary arrangement.

**FIGURE 2:**
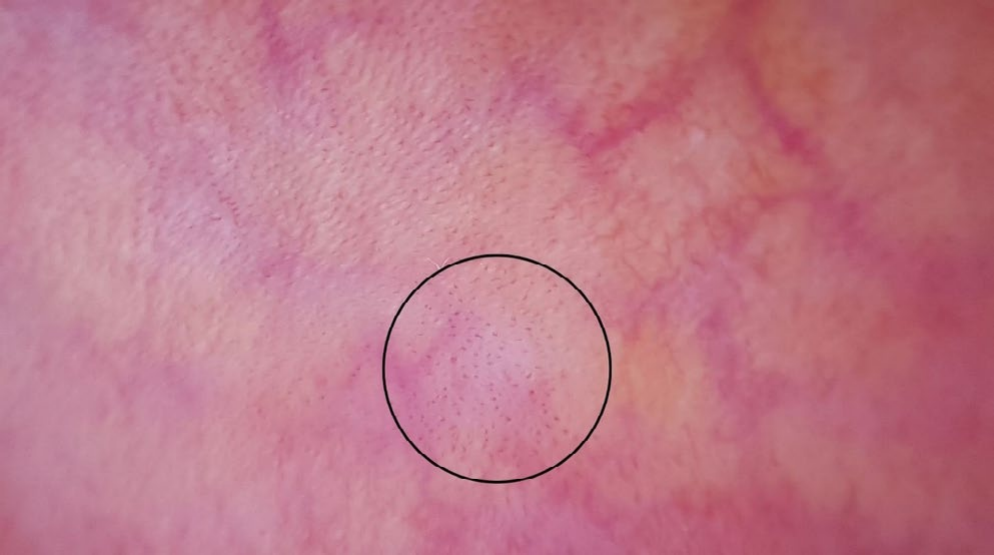
Dot vessels (circled area).

**FIGURE 3:**
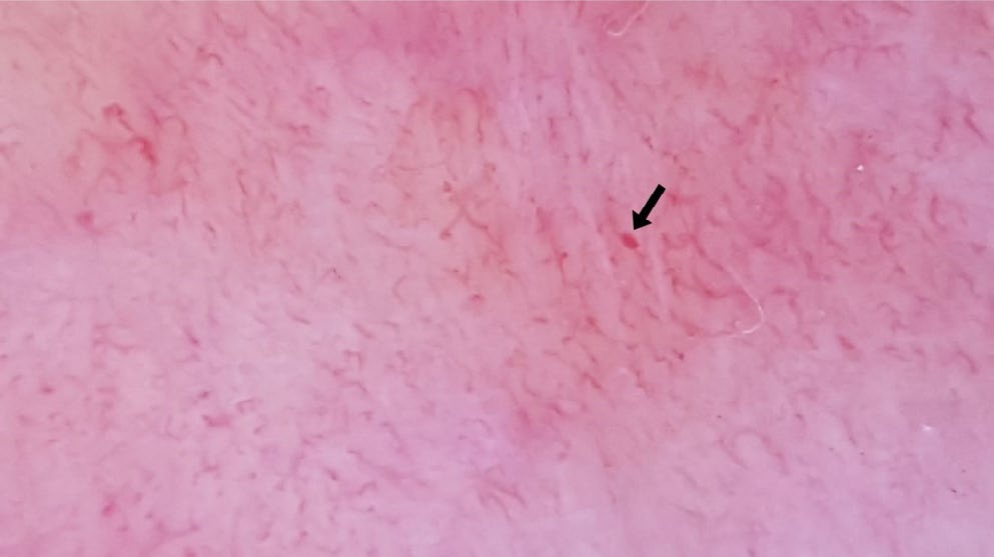
Microhemorrhage (black arrow).

**FIGURE 4:**
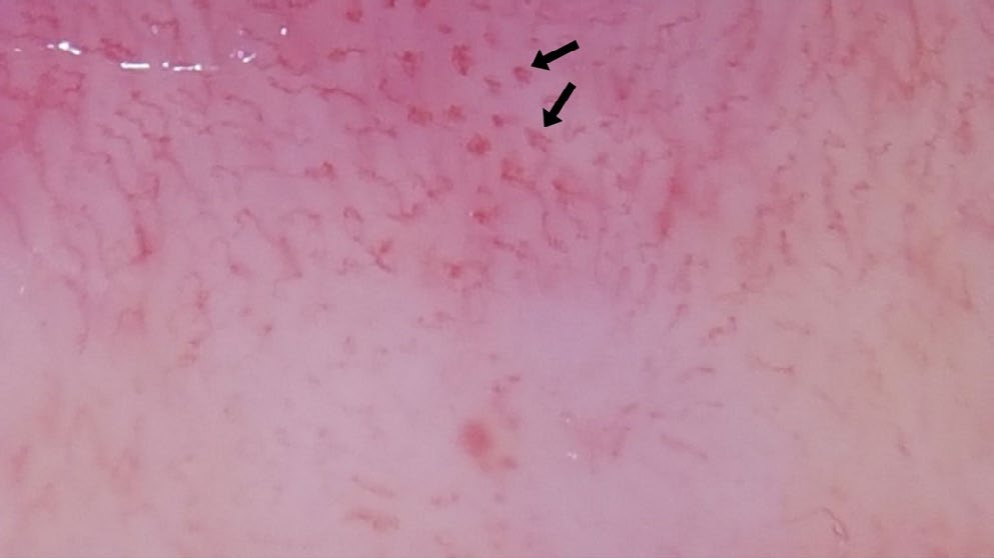
Glomerular vessels (black arrows).

**FIGURE 5:**
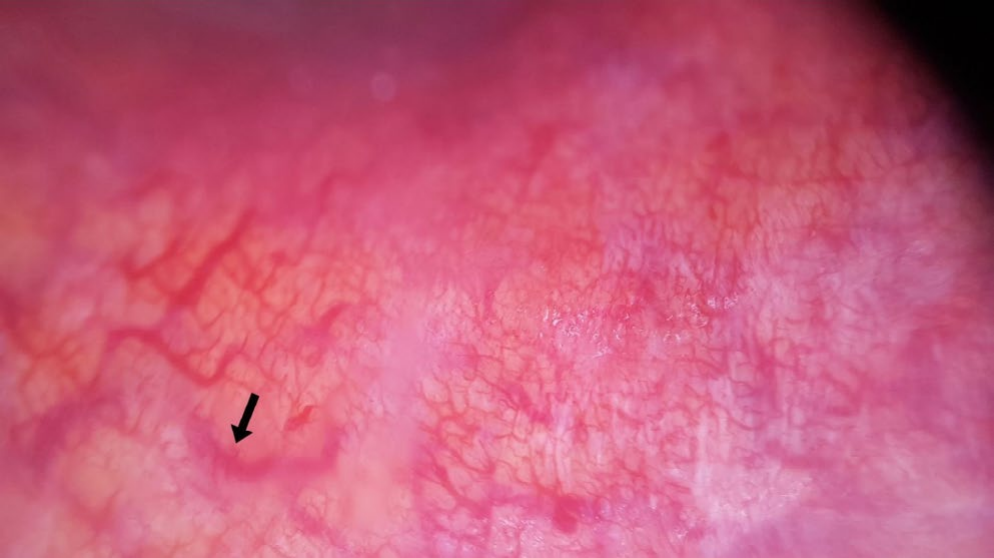
Megacapillaries (black arrow).

**FIGURE 6:**
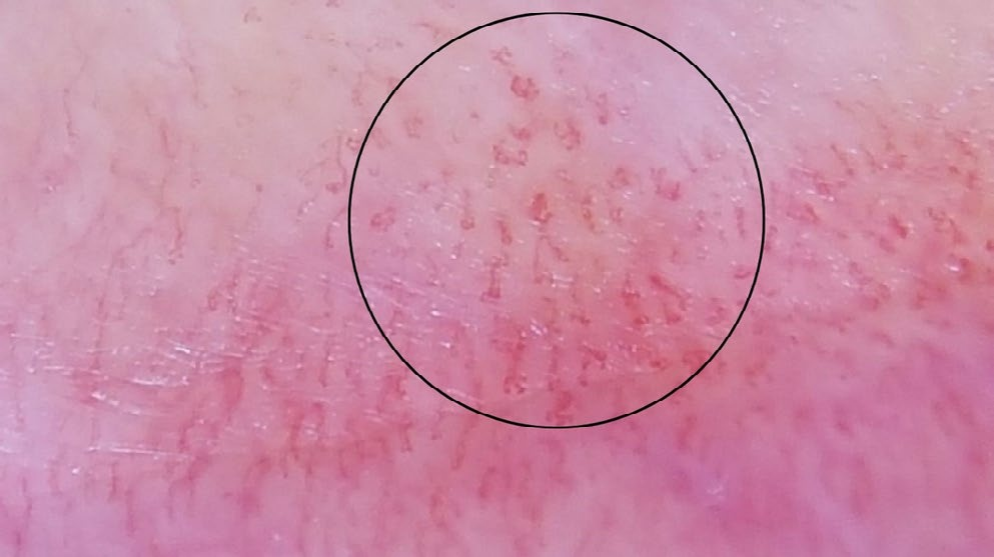
Tortuous capillaries (circled area).

**FIGURE 7:**
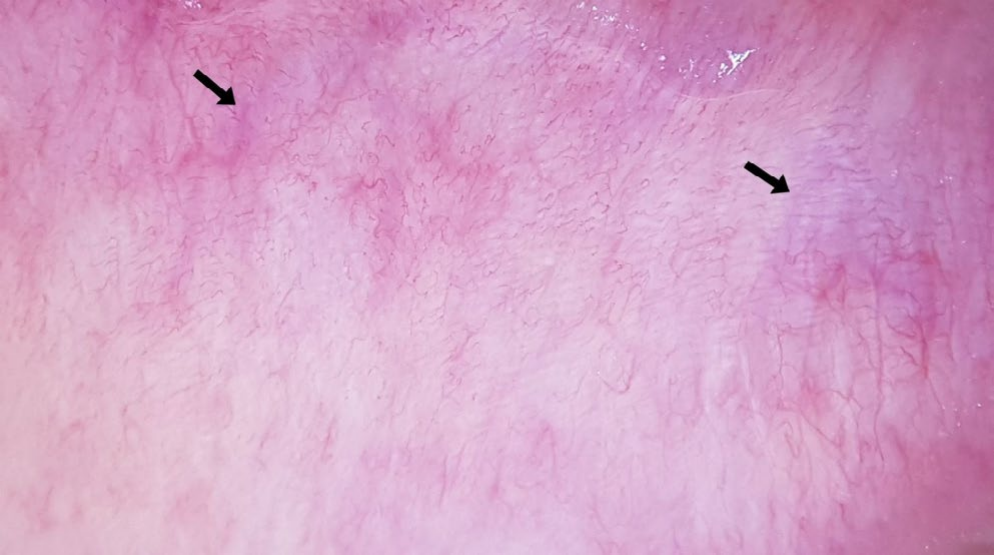
Purple areas (black arrows).

**FIGURE 8:**
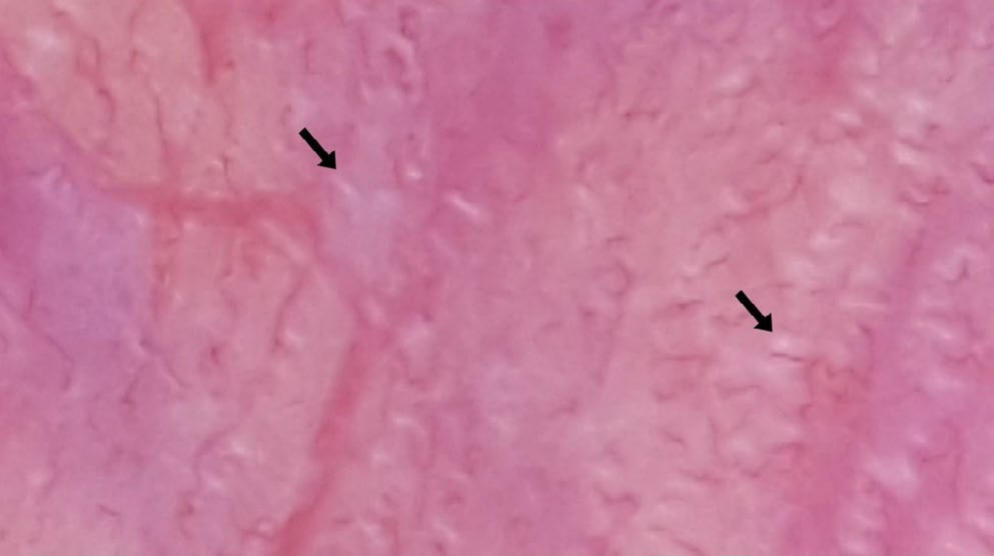
White dots (black arrows).

**FIGURE 9:**
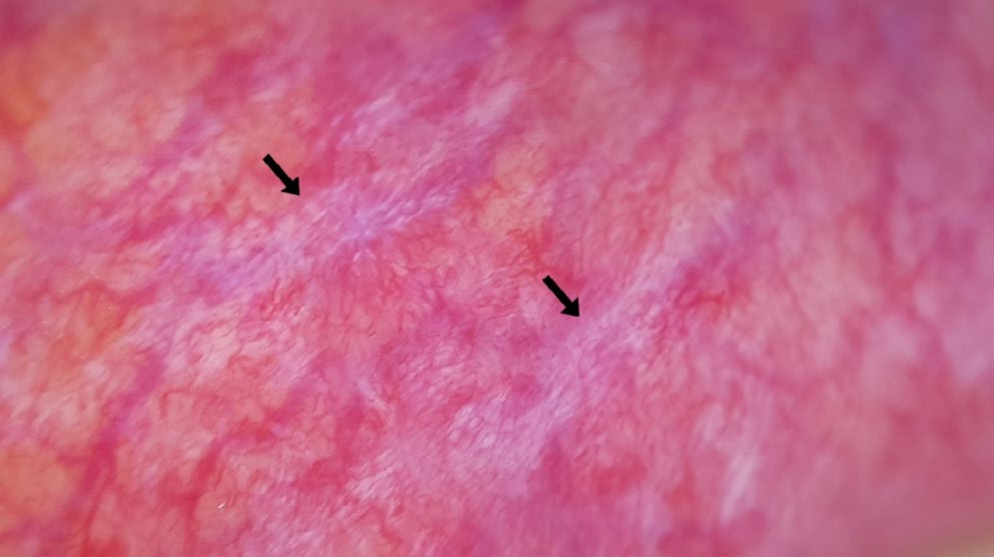
Hyperkeratosis (black arrows).

Oral capillaroscopy was conducted on all participants using a DermLite DL4 handheld dermatoscope with polarized light. Each participant was seated upright in a controlled environment with stable lighting and a room temperature of 23°C–24°C. Images were captured with an iPhone 11 at 20× magnification using 2× optical zoom. Labial mucosa was examined for optimal capillary visibility, and images were analyzed by a trained capillaroscopy specialist to ensure consistency. To maintain proper sterilization, the dermatoscope's contact plate was disinfected using 70% isopropyl alcohol before and after each examination. The evaluated parameters included capillary arrangement (regular or irregular), dot vessels, microhemorrhages, glomerular vessels, megacapillaries, tortuous capillaries, purple areas (indicating blood pooling), white dots (indicating localized tissue changes), and hyperkeratosis (Figures 1, 2, 3, 4, 5, 6, 7, 8, 9). These features were assessed for their prevalence and association with rosacea severity.

## 3 Statistical Analysis

Descriptive statistics, including mean, standard deviation, median, and quartiles, were calculated for all variables. The Kolmogorov–Smirnov test was used to assess the normality of numerical variables. For comparisons between groups, an independent sample *t*‐test was applied to age, while Pearson's chi‐square test evaluated capillaroscopic findings. Associations between disease duration, severity, and capillary findings were analyzed using the Mann–Whitney *U* and chi‐square tests. A significance level of *p* < 0.05 was set for all tests, and analyses were conducted using IBM SPSS Statistics 22.

## 4 Ethics Statement

All the procedures followed the Helsinki declaration, and the study was approved by an Institutional Review Board (Decision date and number: 2020/001). Written informed consent was obtained from all participants.

## 5 Results

A total of 100 rosacea patients and 100 control subjects were included in the study. Gender distribution and mean age were comparable between groups, with no statistically significant differences (*p* = 0.502, *p* = 0.467; Table 1). Oral capillaroscopic findings indicated significantly higher rates of family history of rosacea and tortuous capillaries in the patient group, while other capillaroscopic parameters did not show significant differences (Table 1).

**TABLE 1:** Demographic and capillaroscopic findings in rosacea patients and controls.

Parameter	Category	Control (*n* = 100)	Rosacea (*n* = 100)	*p*
Gender	Male	21	25	0.502
	Female	79	75	
Age (mean ± SD)		43.93 ± 8.78	44.88 ± 9.64	0.467
Disease duration (years)			3.53 ± 2.16	
Fitzpatrick skin type	Type 1	4	9	0.324
	Type 2	28	34	
	Type 3	56	47	
	Type 4	12	10	
Family history	Negative	93	79	**0.004**
	Positive	7	21	
Capillary arrangement	Regular	100	100	—
	Irregular	—	—	
Dot vessels	Absent	64	62	0.770
	Present	36	38	
Microhemorrhage	Absent	80	82	0.718
	Present	20	18	
Glomerular vessels	Absent	86	81	0.341
	Present	14	19	
Megacapillaries	Absent	78	68	0.111
	Present	22	32	
Tortuous capillaries	Absent	70	52	**0.009**
	Present	30	48	
Purple areas	Absent	62	68	0.374
	Present	38	32	
White dots	Absent	82	84	0.707
	Present	18	16	
Hyperkeratosis	Absent	82	85	0.568
	Present	18	15	

*P* < 0.05 is defined statistically significant and shown in bold.

Descriptive statistics on disease duration among rosacea patients revealed that those with positive microhemorrhages had a significantly longer disease duration; other capillaroscopic findings showed no association with disease duration (Table 2).

**TABLE 2:** Disease duration based on capillaroscopic findings in rosacea patients.

Capillary finding	Category	*N*	Mean disease duration (years)	SD	Median	*p*
Capillary arrangement	Regular	200	3.5	2.2	3.0	—
Dot vessels	Negative	126	3.7	2.1	3.0	0.280
	Positive	74	3.3	2.2	2.3	
Microhemorrhage	Negative	162	3.3	2.0	2.8	**0.035**
	Positive	38	4.6	2.7	4.3	
Glomerular vessels	Negative	167	3.6	2.2	3.0	0.565
	Positive	33	3.4	2.2	2.3	
Megacapillaries	Negative	146	3.3	1.8	3.0	0.599
	Positive	54	3.9	2.7	3.5	
Tortuous capillaries	Negative	122	3.8	2.3	3.0	0.207
	Positive	78	3.3	2.0	2.5	
Purple areas	Negative	130	3.7	2.4	3.0	0.579
	Positive	70	3.2	1.6	3.0	
White dots	Negative	166	3.5	2.2	3.0	0.708
	Positive	34	3.6	2.0	3.0	
Hyperkeratosis	Negative	167	3.7	2.2	3.0	0.204
	Positive	33	2.8	1.4	2.3	

*P* < 0.05 is defined statistically significant and shown in bold.

Comparisons of capillaroscopic findings across rosacea subtypes showed no significant difference in tortuous capillary positivity between papulopustular and erythematotelangiectatic types. However, none of the five patients with phymatous rosacea showed positive findings, leading to a significantly lower positivity rate compared to other types. Both patients with ocular rosacea tested positive, but due to the small sample size, further comparisons for the ocular type were excluded (Table 3).

**TABLE 3:** Capillaroscopic findings based on rosacea subtype.

Capillary finding	Subtype	ET (*n*, %)	Phymatous (*n*, %)	Ocular (*n*, %)	Papulopustular (*n*, %)	*p*
Capillary arrangement	Regular	56 (100.0%)	5 (100.0%)	2 (100.0%)	37 (100.0%)	—
Dot vessels	Negative	39 (69.6%)	3 (60.0%)	1 (50.0%)	19 (51.4%)	0.348
	Positive	17 (30.4%)	2 (40.0%)	1 (50.0%)	18 (48.6%)	
Microhemorrhage	Negative	46 (82.1%)	3 (60.0%)	2 (100.0%)	31 (83.8%)	0.540
	Positive	10 (17.9%)	2 (40.0%)	0 (0.0%)	6 (16.2%)	
Glomerular vessels	Negative	48 (85.7%)	3 (60.0%)	1 (50.0%)	29 (78.4%)	0.301
	Positive	8 (14.3%)	2 (40.0%)	1 (50.0%)	8 (21.6%)	
Megacapillaries	Negative	38 (67.9%)	1 (20.0%)	1 (50.0%)	28 (75.7%)	0.086
	Positive	18 (32.1%)	4 (80.0%)	1 (50.0%)	9 (24.3%)	
Tortuous capillaries	Negative	31 (55.4%)	5 (100.0%)	0 (0.0%)	16 (43.2%)	**0.043**
	Positive	25 (44.6%)	0 (0.0%)	2 (100.0%)	21 (56.8%)	
Purple areas	Negative	42 (75.0%)	3 (60.0%)	1 (50.0%)	22 (59.5%)	0.400
	Positive	14 (25.0%)	2 (40.0%)	1 (50.0%)	15 (40.5%)	
White dots	Negative	46 (82.1%)	4 (80.0%)	1 (50.0%)	33 (89.2%)	0.446
	Positive	10 (17.9%)	1 (20.0%)	1 (50.0%)	4 (10.8%)	
Hyperkeratosis	Negative	46 (82.1%)	4 (80.0%)	2 (100.0%)	33 (89.2%)	0.725
	Positive	10 (17.9%)	1 (20.0%)	0 (0.0%)	4 (10.8)	

*P* < 0.05 is defined statistically significant and shown in bold.

In relation to rosacea severity, moderate‐severity patients had a significantly lower rate of microhemorrhage positivity, while the presence of tortuous capillaries increased with severity. No other significant associations were identified between rosacea severity and capillaroscopic findings (Table 4).

**TABLE 4:** Relationships between rosacea severity and oral capillaroscopic findings in rosacea patients.

Capillary finding	Subtype	Rosacea severity			*p*
		Mild (*n*, %)	Moderate (*n*, %)	Severe (*n*, %)	
Capillary arrangement	Regular	22 (100.0%)	50 (100.0%)	28 (100.0%)	—
Dot vessels	Negative	15 (68.2%)	32 (64.0%)	15 (53.6%)	0.526
	Positive	7 (31.8%)	18 (36.0%)	13 (46.4%)	
Microhemorrhage	Negative	17 (77.3%)	47 (94.0%)	18 (64.3%)	**0.004**
	Positive	5 (22.7%)	3 (6.0%)	10 (35.7%)	
Glomerular vessels	Negative	15 (68.2%)	44 (88.0%)	22 (78.6%)	0.132
	Positive	7 (31.8%)	6 (12.0%)	6 (21.4%)	
Megacapillaries	Negative	13 (59.1%)	38 (76.0%)	17 (60.7%)	0.228
	Positive	9 (40.9%)	12 (24.0%)	11 (39.3%)	
Tortuous capillaries	Negative	20 (90.9%)	24 (48.0%)	8 (28.6%)	**0.001**
	Positive	2 (9.1%)	26 (52.0%)	20 (71.4%)	
Purple areas	Negative	12 (54.5%)	38 (76.0%)	18 (64.3%)	0.176
	Positive	10 (45.5%)	12 (24.0%)	10 (35.7%)	
White dots	Negative	17 (77.3%)	44 (88.0%)	23 (82.1%)	0.495
	Positive	5 (22.7%)	6 (12.0%)	5 (17.9%)	
Hyperkeratosis	Negative	17 (77.3%)	43 (86.0%)	25 (89.3%)	0.409
	Positive	5 (22.7%)	7 (14.0%)	3 (10.7%)	

*P* < 0.05 is defined statistically significant and shown in bold.

## 6 Discussion

Rosacea is a chronic inflammatory skin condition which is characterized by facial erythema, telangiectasia, papules, and pustules. This multifactorial condition has an uncertain etiology resulting from a complex interplay of genetic, environmental, and vascular factors [1, 2]. The pathogenesis of rosacea is significantly influenced by various physiochemical factors that stimulate vasodilation and angiogenesis. A key factor in this process is the elevated level of VEGF, which is often stimulated by UV exposure. This increase in VEGF is closely related to endothelial nitric oxide (eNO), a substance that is essential for promoting vasodilation. Furthermore, cathelicidins, particularly the derived peptides like LL‐37, are implicated in both angiogenesis and neovascularization. The changes induced by cathelicidins can further enhance vascularity in rosacea through the stimulation of downstream receptors, such as FPRL1 and EGFR, leading to additional increases in VEGF levels. Consequently, this creates a progressive cycle of chronic vasodilation and angiogenesis that contributes to persistent erythema and the characteristic vascular changes seen in rosacea, including larger and more dilated cutaneous vessels, as well as telangiectasias [3, 4, 5].

Rosacea is classified into four main subtypes, each with unique clinical and pathophysiological traits. The erythematotelangiectatic subtype involves persistent redness and visible blood vessels due to neurovascular dysregulation. The papulopustular subtype, resembling acne, is marked by inflammatory papules and pustules, likely linked to an immune response to Demodex mites. The phymatous subtype, seen as a more severe form, leads to skin thickening and fibrosis, particularly on the nose, due to prolonged inflammation. Lastly, ocular rosacea includes symptoms like dryness, irritation, and blepharitis, potentially sharing inflammatory pathways with cutaneous rosacea [6, 7].

Diagnosis of rosacea is primarily clinical, based on the presence of characteristic signs and symptoms. Routine clinical observation plays a crucial role in diagnosis, as there are no standardized objective methods for assessing disease severity or progression [8].

The potential of noninvasive techniques in dermatology has led to various imaging methods for rosacea, including reflectance confocal microscopy, spectrometry, and transepidermal water loss measurements. Each of these techniques offers insights into skin morphology and function, yet they primarily focus on the skin surface, often overlooking subdermal and mucosal microcirculatory changes [8]. In addition previous investigations into nailfold capillaroscopy in rosacea have yielded controversial results. One study examined nailfold capillary beds in eight patients and matching controls using a stereomicroscope, finding no specific capillaroscopic patterns associated with rosacea [9]. Similarly, another study employing videocapillaroscopy on the cheeks and nail folds of patients with erythematotelangiectatic rosacea (ETR), seborrheic dermatitis, and healthy controls did not reveal differences in the nail folds among the groups. However, notable findings were observed on the cheeks, where patients with ETR displayed significantly larger polygons, more prominent telangiectasia, larger mean vessel diameters, and evidence of neoangiogenesis [10]. A study involved 16 female patients diagnosed with rosacea, revealing atypical nailfold capillaries in all subjects. Common abnormalities included Raynaud loops, meandering capillaries, and an increased number of capillaries compared to healthy controls [11].

Oral capillaroscopy is a technique that allows for direct visualization of the microvascular bed within the mucosal tissues [12]. It has been utilized in various conditions to assess microvascular abnormalities, particularly in autoimmune diseases such as oral lichen planus, Hashimoto's thyroiditis, rheumatoid arthritis, Sjögren's syndrome, systemic sclerosis, and burning mouth syndrome [13, 14, 15, 16, 17, 18, 19, 20]. The absence of the stratum corneum in the oral mucosa allows for clearer visualization of the microvascular bed. Research indicates that the labial mucosa is the best site for oral capillaroscopy due to its visibility and ease of access. The clearer capillary structure in the oral mucosa allows for the observation of certain microvascular changes that are not visible on the skin surface, as well as the impact of VEGF on the disease's pathological processes. This technique can improve our understanding of the causes, treatment, and outlook of different skin conditions [19]. However, there is currently a lack of studies utilizing oral capillaroscopy in rosacea.

This study aimed to investigate the utility of oral capillaroscopy in identifying microvascular changes in rosacea patients. We observed that rosacea patients had significantly higher rates of both family history and tortuous capillaries compared to the control group. While this indicates a potential genetic role in the vascular structures of rosacea, it does not establish a direct causal link. The tortuous capillaries observed in our study may result from chronic inflammation, with prolonged VEGF elevation driving structural capillary remodeling. This key mediator of angiogenesis and vascular permeability likely contributes to endothelial dysfunction and the formation of tortuous vessels, as visualized through oral capillaroscopy. Additionally, our study found that patients with positive microhemorrhages had a longer disease duration, indicating that chronic progression may be linked to increased capillary fragility. This fragility may be attributed to the cumulative effects of inflammation and VEGF‐driven vascular permeability, which weaken capillary walls over time. Microhemorrhages, as observed through capillaroscopy, reflect this vascular instability and provide insight into the progression of rosacea. Such findings suggest that targeting VEGF‐mediated pathways could be an effective strategy for preventing these complications in long‐term patients. These findings suggest that targeting VEGF‐mediated pathways with anti‐inflammatory therapies may help prevent microhemorrhages, highlighting oral capillaroscopy's value in tracking vascular changes and disease progression in rosacea.

The comparison of capillaroscopic patterns across rosacea subtypes showed no significant difference in tortuous capillaries between papulopustular and erythematotelangiectatic types. In contrast, the absence of positive findings in phymatous rosacea suggests that this subtype may involve different, nonvascular mechanisms consistent with its fibrotic nature. While both patients with ocular rosacea showed positive findings, the small sample size limits conclusions about this group. Overall, the findings indicate that vascular changes differ among rosacea subtypes, but no specific capillaroscopic pattern was identified for any of them.

The relationship between rosacea severity and capillaroscopic findings indicates that as severity increases, microhemorrhage positivity decreases, and tortuous capillaries become more common. These changes identified through oral capillaroscopy may serve to evaluate disease severity and provide insights into VEGF‐mediated vascular modifications for personalized treatment approaches.

The limitations of our study are its cross‐sectional design and small sample size for certain subtypes, particularly in ocular rosacea. To further explore the role of vascular changes in the pathophysiology of rosacea, longitudinal studies with larger samples are essential.

This study investigated oral mucosal capillaroscopy in patients with rosacea, highlighting the potential for this noninvasive method to detect microvascular abnormalities associated with the disease. The distinct vascular changes observed in different rosacea subtypes highlight the importance of personalized management approaches. Our findings suggest that oral mucosal capillaroscopy could serve as a valuable tool for monitoring vascular changes, disease progression, and possibly therapeutic response.

## Ethics Statement

The study received approval from the institutional ethics committee (Decision Number: 2020/001). Written informed consent was obtained from all participants prior to study enrollment.

## Conflicts of Interest

The authors declare no conflicts of interest.

## Data Availability

The data that support the findings of this study are available from the corresponding author upon reasonable request.
